# Acoustic Cell Patterning for Structured Cell‐Laden Hydrogel Fibers/Tubules

**DOI:** 10.1002/advs.202308396

**Published:** 2024-02-02

**Authors:** Qiu Yin, Yucheng Luo, Xianglin Yu, Keke Chen, Wanlu Li, Hu Huang, Lin Zhang, Yinning Zhou, Benpeng Zhu, Zhichao Ma, Wenming Zhang

**Affiliations:** ^1^ State Key Laboratory of Mechanical System and Vibration Shanghai Jiao Tong University Shanghai 200240 China; ^2^ Institute of Medical Robotics, School of Biomedical Engineering Shanghai Jiao Tong University No.800 Dongchuan Road Shanghai 200240 China; ^3^ SJTU Paris Elite Institute of Technology Shanghai Jiao Tong University Shanghai 200240 China; ^4^ School of Biomedical Engineering and Med‐X Research Institute and Shanghai Jiao Tong University Shanghai 200030 P. R. China; ^5^ Key Laboratory of CNC Equipment Reliability, Ministry of Education, School of Mechanical and Aerospace Engineering Jilin University Changchun Jilin 130022 China; ^6^ School of Mechatronic Engineering Changchun University of Technology Changchun 130012 China; ^7^ Joint Key Laboratory of the Ministry of Education, Institute of Applied Physics and Materials Engineering University of Macau, Avenida da Universidade Taipa, Macau 999078 China; ^8^ School of Integrated Circuit, Wuhan National Laboratory for Optoelectronics Huazhong University of Science and Technology Wuhan 430074 China

**Keywords:** acoustofluidic, biofabrication, cell patterning, hydrogel fibers

## Abstract

Cell‐laden hydrogel fibers/tubules are one of the fundamentals of tissue engineering. They have been proven as a promising method for constructing biomimetic tissues, such as muscle fibers, nerve conduits, tendon and vessels, etc. However, current hydrogel fiber/tubule production methods have limitations in ordered cell arrangements, thus impeding the biomimetic configurations. Acoustic cell patterning is a cell manipulation method that has good biocompatibility, wide tunability, and is contact‐free. However, there are few studies on acoustic cell patterning for fiber production, especially on the radial figure cell arrangements, which mimic many native tissue‐like cell arrangements. Here, an acoustic cell patterning system that can be used to produce hydrogel fibers/tubules with tunable cell patterns is shown. Cells can be pre‐patterned in the liquid hydrogel before being extruded as cross‐linked hydrogel fibers/tubules. The radial patterns can be tuned with different complexities based on the acoustic resonances. Cell viability assays after 72 h confirm good cell viability and proliferation. Considering the biocompatibility and reliability, the present method can be further used for a variety of biomimetic fabrications.

## Graphical abstract

An acoustofluidic system that can be used to produce hydrogel fibers/tubules with tunable cell patterns is proposed. Cells can be pre‐patterned in the liquid hydrogel before being extruded as cross‐linked hydrogel fibers/tubules. The radial patterns can be tuned with different complexities based on the acoustic resonances.

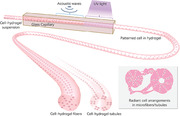

## 1 Introduction

Bio‐fabrication seeks to create synthetic biological substitutes to replicate the structural and physicochemical features of natural tissues. This endeavor is important in various fields, such as food production,^[^ ^1^ ^]^ regenerative medicine^[^ ^2^ ^]^ and drug discovery.^[^ ^3^ ^]^ Consequently, the ability to produce on‐demand biocompatible scaffolds with controllable cell distribution emerges as one of the essential techniques in bio‐fabrication.

In recent years, hydrogel fibers have played an important role in fulfilling the above requirements as biocompatible tissue scaffolds.^[^ ^4^, ^5^, ^6^ ^]^ It holds great promise for constructing tissue‐like structures with topographical properties that vary spatiotemporally at multiple scales.^[^ ^7^ ^]^ Moreover, the hydrogel fibers can include various biomolecules and microorganisms to support their functions.^[^ ^8^ ^]^ However, the random cell encapsulation in hydrogel fibers commonly leads to isotropic cell organization, in contrast to the natural cell anisotropy in many natural tissues. This mismatch might challenge the biomimicry of the engineering tissue structures, which play a great role in embryogenesis,^[^ ^9^ ^]^ tissue maturation,^[^ ^10^ ^]^ and regeneration growth.^[^ ^11^ ^]^ For example, researches show the facts that cell alignment affects neuron regeneration,^[^ ^10^, ^12^ ^]^ or modulates the mechanical properties of tissues including the skeleton,^[^ ^13^ ^]^ cardiac muscle,^[^ ^14^ ^]^ and tendon.^[^ ^15^ ^]^ In addition to the parallel lines of cells in the tissue, studies have shown that there are other shapes of cell arrangements. For example, there are some radiant structures surrounding the vessels.^[^ ^16^ ^]^ Besides, in one of the basic liver microtissues, hepatic lobules, the radiant patterns of hepatocytes promote liver functionality.^[^ ^11^ ^]^

To address the challenge of constructing biomimetic structures, the ability to form cell patterns within hydrogel fibers will allow strategies to engineer the cellular architecture and function of the organisms. For example, Norotte and Skardal et al.^[^ ^17^, ^18^ ^]^ showed a method for making tubular vascular that can form different patterns. They used the agarose rods as a molding template and then assembled tissue spheroids into customized tubular structures with defined topology. Besides, the development of microfabrication (e.g., lithography^[^ ^19^, ^20^ ^]^ micro‐machining,^[^ ^21^ ^]^ and 3D printing^[^ ^22^, ^23^, ^24^, ^25^ ^]^), micro‐molds have been used for guiding cell network proliferation. In addition, there are many approaches to manipulating cells using external physical fields to improve efficiency, countability, and flexibility, including gravitational,^[^ ^26^ ^]^ electrical,^[^ ^27^ ^]^ magnetic,^[^ ^28^, ^29^ ^]^ and acoustic fields.^[^ ^30^, ^31^ ^]^ These approaches provide active control of cellular patterning and also validate the importance of native cellular architecture for tissue function.

Among these various external fields, the acoustic methods show the advantages of good compatibility, fast response, contact‐free, and widescale tunability, thus innovating the strategies of cell patterning.^[^ ^32^, ^33^ ^]^ The acoustofluidic configurations can be easily integrated with a microfluid environment, and avoid the effort for magnetic or electrical labeling.^[^ ^34^, ^35^ ^]^ For example, Bouyer et al.^[^ ^36^ ^]^ developed a Bio‐Acoustic Levitational (BAL) assembly device to generate 3D multilayered neural tissue to study the acoustic cell patterns for neural differentiation, which is a simple, biocompatible platform to create physiologically relevant in vitro neuronal models of the brain, including the multilayered cortex or the cerebellum. Armstrong et al.^[^ ^37^ ^]^ used standing acoustic waves to pattern myoblasts in gelatin methacryloyl hydrogel. The results showed that parallel patterning of myoblasts exhibited the enhancement of myofibrillogenesis and promoted the formation of muscle fibers containing aligned bundles of myotubes. Besides, the acoustic assembled organoids also present good proliferative ability^[^ ^38^ ^]^ Moreover, the subsequent cell spreading and migration will not affect the cell patterns.^[^ ^37^, ^38^ ^]^ Kang et al.^[^ ^39^ ^]^ proposed an acoustophoretic approach to fabricate implantable 3D‐patterned collateral microvessels for improving ischemia. Furthermore, to form complex patterns, the scientists also developed acoustic holography for complex‐shaped cell patterning and assembly.^[^ ^40^, ^41^ ^]^

Deshmukh and co‐workers^[^ ^42^ ^]^ demonstrated a method based on acoustically activated capillaries for generating parallel cell bundles within hydrogel fibers, which paves the way for continuous production of structured cell‐laden hydrogel fibers. There is another acoustic‐based cell patterning work by Le et al.,^[^ ^43^ ^]^ who used acoustic standing waves to assemble human umbilical vein endothelial cells into parallel lines to form 3D micro‐vascular networks. In terms of mimicking the micro‐tissue structures, parallel‐line cell patterning might be used in vessel construction, however, it lacks flexibility in shapes (e.g., radiant structures surrounding the vessels.^[^ ^16^ ^]^ There is still a lack of a method that can be used to generate radially arranged cell patterns during fiber production. Recent studies of acoustic resonance in tubes show the possibilities of acoustic cell patterning for producing structured cell‐laden hydrogel fibers.^[^ ^44^, ^45^ ^]^

In this study, we leveraged the advantages of acoustic cell patterning to engineer an acoustofluidic system for bio‐structure‐mimetic hydrogel fibers/tubules production. The cells or microparticles that are suspended in the pre‐cross‐linked hydrogel can be acoustically arranged in different shapes based on the different resonance modes. This enables us to form cell or microparticle pre‐patterns, after which the cross‐linking of the hydrogel scaffold maintains the cell patterns to form structured cell‐laden fiber/tubules. The cell patterns are tunable based on the acoustic frequencies. For example, we demonstrated the formation of 2/4/6‐petal radiant cell assemblies at the corresponding resonance frequencies of the system. Furthermore, we tested the viability of cell‐laden hydrogel fiber/tubules over 72 h, which maintains good cell viability. Our method can form bio‐mimetic cell structures in a hydrogel environment, which has great potential for creating synthetic biological substitutes for structural and physicochemical features of natural tissues.

## 2 Results and Discussion

### 2.1 The Conception of Hydrogel Fiber/Tubule Production with Acoustic Cell Patterning

**Figure 1:**
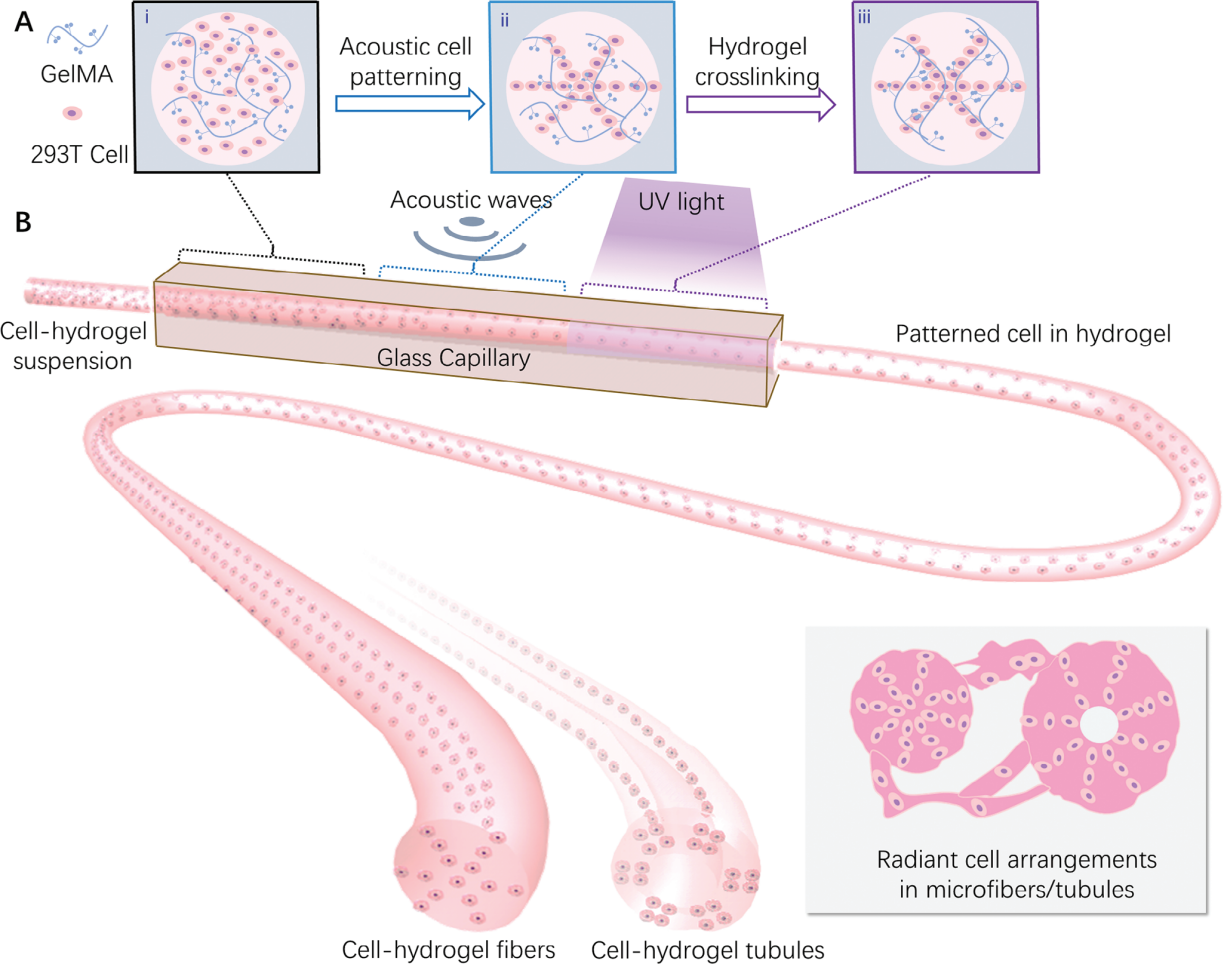
Schematic of the hydrogel fiber/tubule production with acoustic cell patterning. A) (i) Cells are randomly distributed in a liquid hydrogel solution when injected into the capillary. (ii) The designed acoustic pressure fields enable cell patterning in the liquid hydrogel. (iii) The UV light irradiates the liquid hydrogel with cell patterns, within which the photo‐initiators cross‐link the polymer networks, thus the liquid hydrogel will be cured. B) Continuous flow of cell‐laden hydrogel is driven by a syringe pump to the transparent glass capillary. When the flow passes through the acoustic‐activated capillary, the cells will be assembled to the desired pattern. After that, the structural hydrogel fibers/tubules are generated with the cross‐linking by ultraviolet (UV) light.

The conception of acoustically producing structured cell‐laden hydrogel fibers/tubules is shown in **Figure** **1**. The system consists of simplex and commercially available components: a glass capillary, a piezoelectric transducer, and a UV light source. As shown in Figure 1A, the pre‐cross‐linked cell‐hydrogel suspension is injected into the glass capillary with a syringe pump. The capillary provides the container for acoustic cell patterning and the hydrogel cross‐section shaping. In the case of extruding hydrogel tubules, a co‐axial rod is inserted in the center of the capillary as a mold. To increase the contact between the piezoelectric transducer and the glass capillary, the outer boundary of the capillary is cast in a rectangular shape. On the outer boundary of the capillary, the piezoelectric transducer is attached for generating the acoustic waves and excites the resonance of the glass capillary cavity. Upon different acoustic frequencies, there will be different resonance modes for the capillary. As the different resonance modes generate an acoustic pressure distribution at the cross‐sections of the capillary, there will be acoustic radiation force (ARF) acting on the suspended particles or cells in liquid hydrogel to form the patterns corresponding to the acoustic pressure. ARF is the time‐averaged force on a particle suspended in fluid when it is exposed to an acoustic field. The UV light source is for cross‐linking the hydrogel suspension containing gelatin methacryloyl (GelMA), photoinitiator, and particles or cells.

### 2.2 Generation of Radiant Patterned Acoustic Fields Upon Different Resonance Modes

#### 2.2.1 Design of the Capillaries with Different Shapes and Materials

**Figure 2:**
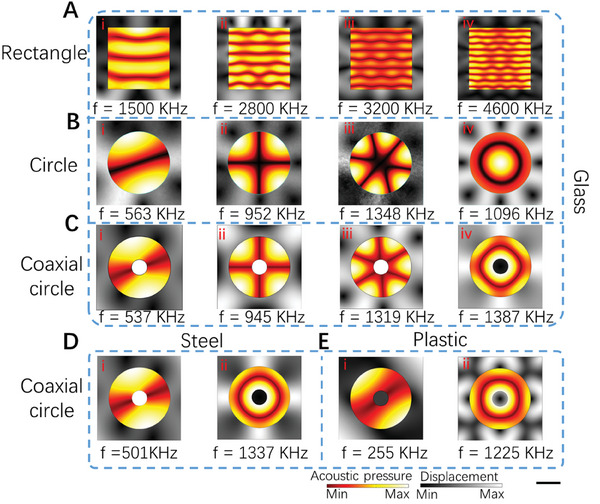
The numerical simulation results of acoustic pressure fields in capillaries with different shapes and materials. The acoustic pressure field distribution in the glass capillary. with different shapes of different inner cross‐sections: A) rectangle capillary. B) circle capillary. and C) coaxial circle capillary. The acoustic pressure field distribution in the coaxial circle capillary. with different materials of D) steel and E) plastic. Scale bar: 500 µm.

As aforementioned, the capillary is one of the major components in our system, and it should be excited with the radiant acoustic field for patterning the cells. Thus, we numerically verified several capillary materials and shapes that are commonly exploited in bio‐fabrication.^[^ ^46^, ^47^ ^]^ Their materials are respectively glass, steel, and plastic. The shapes of the inner cross‐sections include rectangles, circles, and coaxial circles. We numerically simulated the acoustic pressure fields of these capillaries under different conditions (More details see the Materials and Methods). First of all, we studied the rectangle shape for different working frequencies as shown in **Figure** **2**A, we can find that the acoustic pressure field is evenly distributed at a specific distance parallel to the bottom surface, which is similar to the recent work made by Deshmukh et al.^[^ ^42^ ^]^ And as the working frequency increases, the distance will become smaller. However, as shown in Figure 2B, we find that a complex and adjustable acoustic pressure field can be generated inside the circular capillary. It not only produces the radiant‐structural acoustic field in Figure 2B i‐iii, but also the annular pattern in Figure 2B(iv). Most interesting, as shown in Figure 2C, when we insert a microrod to form a coaxial circular capillary, it also shows a similar acoustic pressure field. Moreover, we found that circular capillaries of various materials can have similar acoustic pressure field distribution patterns as shown in Figure 2C–E. It is shown that our method is applicable to multiple materials that are commonly used in bio‐fabrication, such as metal, plastic, and glass. In this article, in order to facilitate the observation of particle movement in the fluid channel during the experiment, we selected the glass circular capillary. as the experimental device considering the excellent optical transparency of glass.

#### 2.2.2 Acoustic Patterning of Microparticles in the Glass Capillary

**Figure 3:**
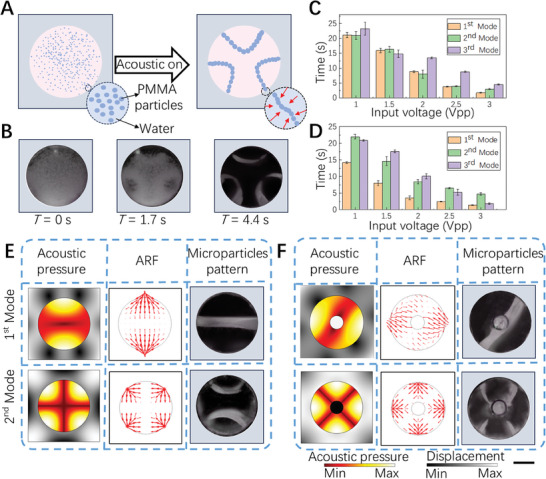
The principle of acoustically controlled particle pattern in a glass capillary. A) The schematic diagram of PMMA particles patterning process under acoustic on. B) The corresponding PMMA particles dynamic patterning experiment after under acoustic at 0, 1.7, and 4.4 s. C) The effect of input voltage on the patterning velocity of particles in glass capillaries under different acoustic modes. D) The effect of the input voltage on the patterning velocity of particles in coaxial glass capillaries under different acoustic modes. E) The simulation results of acoustic pressure field and ARF distribution in glass capillaries as well as the corresponding experiment results of particle patterns for different acoustic modes. F) The simulation results of acoustic pressure field and ARF distribution in coaxial glass capillaries as well as the corresponding experiment results of particle patterns for different acoustic modes. Scale bar: 500 µm.

After the investigation of the capillary materials and shapes, we looked into the working principle of acoustic patterning of microparticles in glass capillary. The experiments validated the simulation results via acoustic patterning of polymethylmethacrylate (PMMA) microparticles with a diameter of 20 µm (**Figure** **3**A,B). As shown in Figure 3E, we find that various acoustic resonant modes on a cross‐section of the capillary can be stimulated by changing the input frequency. According to the numerical simulation results of the acoustic pressure field, the pressure nodes represented in the region are located between two high acoustic pressure regions. Furthermore, we also studied the ARF distribution by numerical simulation. According to the ARF calculation formula in numerical simulation, the ARF is dependent on the relative density and sound speed of the particle to the medium, which can be described as an acoustic contrast factor. When the acoustic contrast factor is positive, usually the case where the particles have higher density and sound speed than the medium, the particles will move to pressure nodes where the acoustic pressure is minimal. In contrast, when the acoustic contrast factor is negative, the particles will move to antinodes where acoustic pressure is maximal. In the case of PMMA particles and cells, the acoustic contrast factor is both positive,^[^ ^33^ ^]^ so that, they will be pushed to the pressure nodes due to the ARF. Similarly, we also find that the structure of micro rods inserted in the center of the capillary which is used for microtubule fabrication possesses a similar phenomenon (Figure 3F). In addition, the effect of input voltage on particle patterning speed was also experimentally studied. The results in Figure 3C,D show that for different acoustic modes, a larger input voltage will lead to an increase in acoustic intensity and impose stronger ARF on the particles. Therefore, this will result in faster patterning speed. In particular, when we set the input voltage to 3 Vpp, the pattern formation time is less than 5 s. The results also indicate that the increase of Gelma concentration slows down the cell arrangement process (Figure S1).

### 2.3 Continuous Production of Fibers and Tubules with Patterned Particles

**Figure 4:**
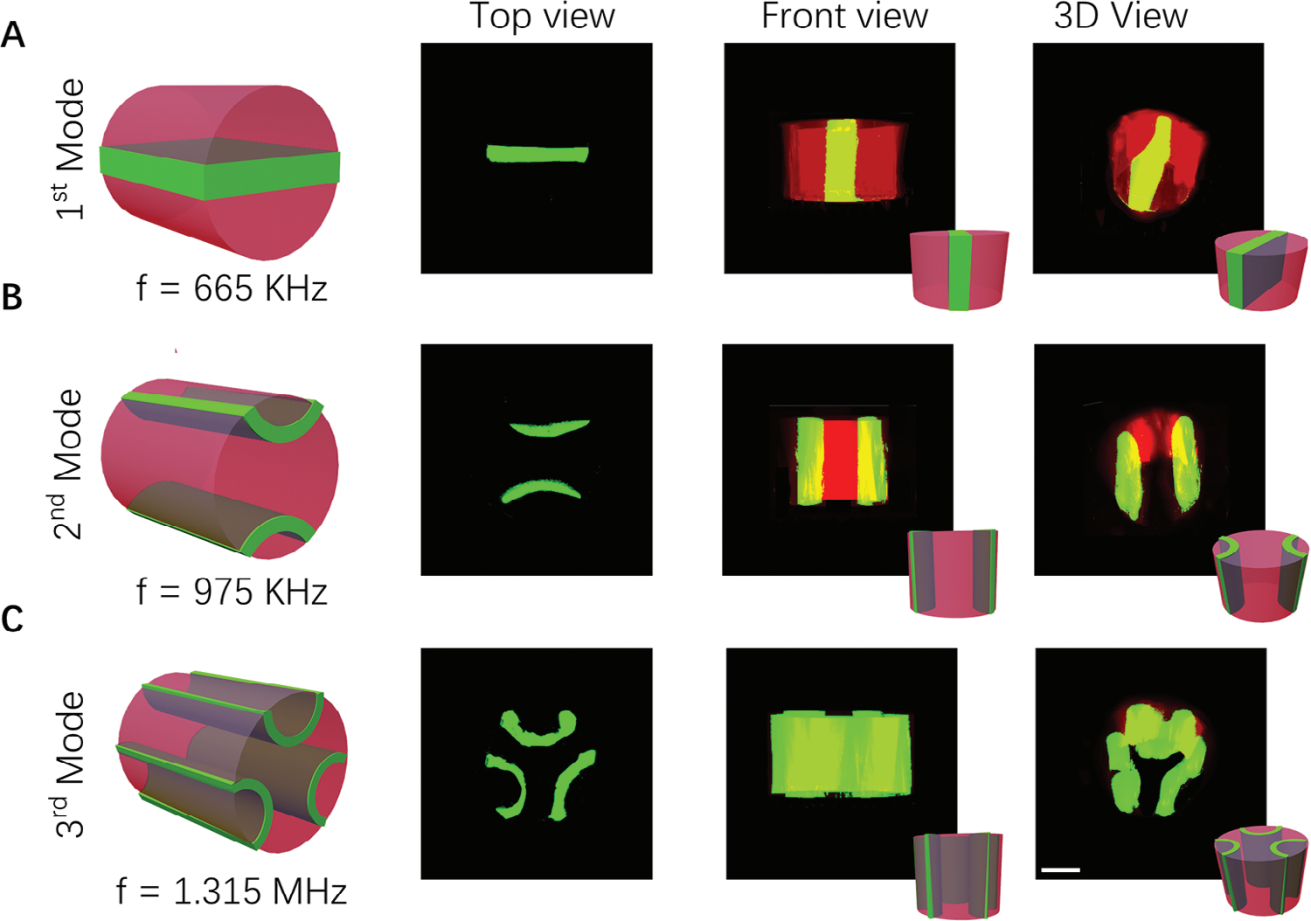
Results of continuous production of hydrogel fibers with patterned fluorescent PS particles. A–C) The different structures (Green: PS particles, Red: Hydrogel) under various acoustic modes (first mode, second mode, third mode) from different views (Left: Top view, Middle: Front view, Right: 3D view). Scale bar: 500 µm.

After studying the principle of acoustically controlled particle patterns in glass capillaries, we first tried to fabricate the hydrogel fibers with patterned particles (**Figure** **4**). During the experiment, we selected a mixture of GelMA hydrogel (commonly used to make tissue culture scaffolds) and Polystyrene (PS) fluorescent particles as raw materials. After the acoustic‐activated microfluidic bioprinting process, as can be seen from the top view, three types of hydrogel fibers with the desired PS fluorescent particles (green) patterns were prepared, corresponding to three different operating frequencies of 665 kHz, 975 kHz, and 1.315 MHz respectively. Besides, to explore the particle patterning effects in the fiber axial direction, we also characterized the produced fibers in front view, the results showed that the particles were aligned in a continuous straight line in the axial direction. Furthermore, we demonstrated the prepared fibers from a 3D view which consists of the cured hydrogel (red) and PS particles (green). The microparticle assembly showed a radiant pattern at the cross‐sections and the hydrogel maintained the assembly after extrusion from the capillary.

**Figure 5:**
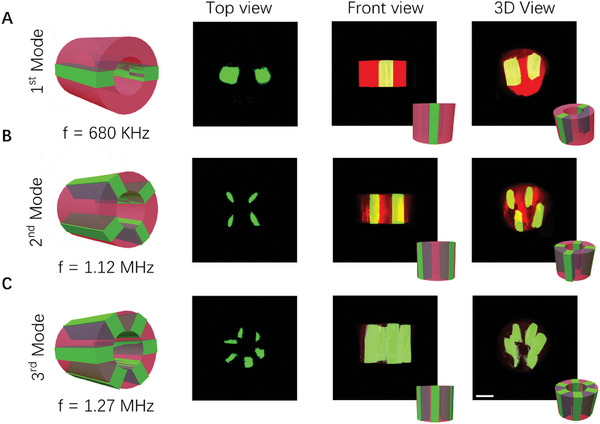
Results of continuous production of hydrogel tubules with patterned fluorescent PS particles. A–C) The different structures (Green: PS particles, Red: hydrogel) under various acoustic modes (first mode, second mode, third mode) from different views (left: top view, middle: front view, right: 3D view). Scale bar: 500 µm.

Besides making hydrogel fibers with patterned particles, we also designed a system for producing hydrogel tubules with patterned particles (**Figure** **5**). As shown in the top view, when operating frequencies of 680 kHz, 1.1 MHz, and 1.33 MHz are used respectively, the particles will be evenly arranged in 2, 4, and 6 lines along the circumferential direction. Besides, the results of the front view showed that the particles are arranged in a continuous straight line in the axial direction. Furthermore, we demonstrated the prepared fibers from a 3D view which consists of the cured hydrogel (red) and PS particles (green).

### 2.4 Continuous Production of Fibers and Tubules with Patterned Cells

**Figure 6:**
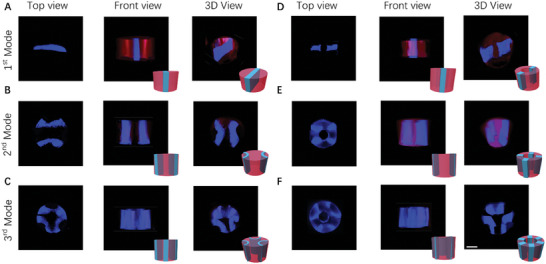
Results of continuous production of hydrogel fibers and tubules with patterned cells. A–C) The different structures (Blue: 293T cell, Red: Hydrogel) under various acoustic modes (first mode, second mode, third mode) from different views (left: top view, middle: front view, right: 3D view) of hydrogel fibers. D–F) The different structures (blue: 293T cell, red: hydrogel) under various acoustic modes (first mode, second mode, third mode) from different views (left: top view, middle: front view, right: 3D view) of hydrogel tubules. Scale bar: 500 µm.

Based on the fabrication of the hydrogel fibers with patterned particles, we investigated the ability of our system to produce fibers and tubules with patterned cells using 293T cells. As shown in **Figure** **6**A–C, three types of hydrogel fibers with the desired cells (blue) patterns are prepared, corresponding to three different operating frequencies. And cells also were arranged in a continuous straight line in the axial direction. similarly, we also fabricated three different hydrogel tubules as shown in Figure 6D–F. These results indicated that it is an ideal method for preparing biomimetic structures.

**Figure 7:**
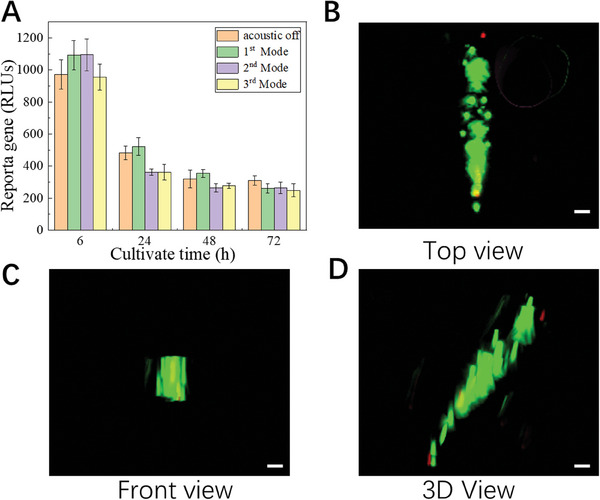
Cell viability tests show that cells grow and proliferate in the acoustically patterned cell‐laden hydrogels. A) The results of the cell toxicity test for four different samples treated with different acoustic conditions (acoustic off, first mode, second mode, third mode) at 6, 24, 48, and 72 h indicate the normal proliferation of viable cells. B–D) Fluorescence images of acoustically patterned 293T after 24 h from different views (B: top view, C: front view, D: 3D view), which demonstrate that cells survive in the acoustically patterned assemblies in the 3D hydrogels, the green region represents the live cells, the red region represents the dead cells. Scale bar: 100 µm.

Furthermore, to verify the biocompatible of our method, the cell toxicity test was conducted. The test is based on the bioluminescent measurement of adenylate kinase (AK) which is present in all cells. When the plasma membrane is damaged and cell integrity is lost, some factors from cultured cells leak into the surrounding medium. We measure the light intensity to determine the cell cytotoxicity and cytolysis because the emitted light intensity is linearly related to the AK concentration. From **Figure** **7**A, we can find that there is no significant difference between the samples without acoustic treatment and the samples with acoustic treatment. Besides, the relative light unit (RLU) is decreased with the increase of cultivation time indicating the normal proliferation of viable cells. Finally, to investigate if the patterned cells survive in the curved hydrogel environment over a long time, a live/dead viability assay was used for imaging in the 3D hydrogel. As shown in Figure 7B–D, a strong green fluorescence signal in the patterned region proves that cells survive in the acoustically patterned assemblies in the 3D hydrogels after 24 hcultivation. Figure S2 shows that the temperature of the system is maintained at around 35 ℃ in the operation conditions.

## 3 Conclusion

In summary, we have demonstrated an acoustofluidic system that can be used to produce hydrogel fibers/tubules with desired cell patterns. Compared to conventional ways, our method permits the assembly of custom‐designed, bio‐mimic shaped cell patterns by controlling the acoustic modes. The pre‐cross‐linked hydrogel suspension containing cells or particles is injected via a micropump, cells or particles can be acoustically arranged in different shapes based on the different resonance modes under the action of ARF. This enables us to form cell or microparticle pre‐patterns, after which the UV‐cured hydrogel scaffolds maintain cell patterns to form structured cell‐laden fiber/tubules. The cell patterns are controllable and adjustable based on the acoustic frequencies. Based on the system, we demonstrated the formation of 2/4/6‐petal radiant cell assemblies at the corresponding resonance frequencies. Furthermore, the cell laden hydrogel fiber/tubules were also proved to show good cell viability over 72 h culture. By considering the safety, biocompatibility and reliability of this method, we think it is suitable for bioprinting. Moreover, because of the importance of bio‐mimic fiber and tubule in tissue scaffolds, our method can be integrated into the 3D bioprinting system to create biocompatible 3D scaffold with controlled architecture and cell distribution in the future.

## 4 Experimental Section

### Device Fabrication

To fabricate the acoustofluidic system, a PZT transducer (Pz4, COSSON) was attached to one side of glass capillary at first. the glass capillary had outer dimensions of 2.2 × 2.2 mm, an inner diameter of 1.6 mm, and a length of 50 mm (for the tubule production system, integrated a microrod with a diameter of 0.4 mm in the center of the glass capillary). Then, the entrance of the capillary connected the syringe pump (LSP01, Longer) with a polyethylene tube with a flow rate of 20 µL min^−1^. To actuate the transducer, a signal generator (DG1022Z, RIGOL) was used and connected to a power amplifier (WMA‐300, Falco System). To avoid the adhesion of uncured GelMA to the glass capillary, a sufficient UV exposure dose (*λ* = 405 nm, intensity = 30 mw cm^−2^) was used to fully cure the hydrogel for 15 s. Thus, the cured GelMA did not adhere to the capillary and can be extruded as fibers/tubules.

### Numerical Simulation

In order to explore the principle of acoustically controlled particle patterns in glass capillaries the numerical modeling of acoustic pressure field was conducted with the FEM software COMSOL Multiphysics, similar to a previously published method.^[^ ^48^ ^]^ To simplify the calculation, a 2D model was used. When calculating the circular tube, the structure consisted of an outer rectangle with a size of 2.2 × 2.2 mm and an inner circle with a diameter of 1.6 mm. Besides, a concentric circle with a diameter of 0.4 mm was added to study the coaxial circle tube. And the circle was set as the acoustic‐structure boundary. The Pressure Acoustics module and the Solid Mechanics module from COMSOL were employed to solve the eigenfrequency and the acoustic pressure field in the capillary. From such computation, the different resonant modes and their corresponding acoustic fields and solid displacements were acquired. Then, the Gor'kov potential distribution *U*_G_ was obtained from the acoustic pressure field.^[^ ^49^ ^]^

(1) $$U_{G}=2\pi R^{3}\left[\frac{\langle \rho^{2}\rangle }{3}\left(\frac{1}{\rho_{m}c_{m}^{2}}-\frac{1}{\rho_{c}c_{c}^{2}}\right)-\left(\frac{\rho_{c}-\rho_{m}}{2\rho_{c}+\rho_{m}}\right)\rho_{m}\langle v^{2}\rangle \right]$$

where R is the radius of the computation cell, 〈*p* ^2^〉 and 〈*v* ^2^〉 are respectively the mean‐square fluctuations of the acoustic pressure and velocity, *ρ*_c_ and *ρ*_m_ are the density of the computation cell and medium, respectively, *c*_c_ and *c*_m_ are the speed of sound of the computation cell and medium, respectively. Thus, the acoustic radiation force (*F*_R_) can be calculated as

(2) $$F_{R}=-\nabla U_{G}$$

### Preparation of the Liquid Hydrogel with Cells (Particles) Mixture

Cells were suspended in gelatin methacrylate (GelMA, EFL‐GM‐60, Suzhou, China) solution, which was prepared by using 0.25 wt.% lithium phenyl‐2,4,6‐trimethylbenzoylphosphinate (LAP) photoinitiator to dissolve 5.6 wt.% GelMA powder and 2 wt.% Pluronic F‐127. After heated at 50 °C for ≈30 min in a water bath, the GelMA solution was prepared, and all the procedures needed to be conducted in the dark. 293T human embryonic kidney cells were used in all cell patterning experiments. They were cultured on a plastic petri dish with DMEM high sugar complete medium (SNM‐002E, SUNNELL) in a 5% CO_2_ incubator at 37 °C before use. For experimental use, the cultivated cells were washed twice with phosphate‐buffered saline (PBS, Adamas‐Life) solution and were detached from the substrate using 1 mL 0.125% trypsin (Invitrogen, USA) for 1 min. Then, the trypsinization process was stopped by adding 2 mL of fresh cell culture medium. The detached cells were centrifuged at 1000 rpm for 5 min and resuspended in PBS solution; the concentration was maintained at 5 × 10^7^ cells mL^−1^. For the cell mixture preparation, 100 µL of 5 × 10^7^ mL^−1^ cell suspension was added to 900 µL GelMA solution prepared before, then the hydrogel mixture with 5% GelMA and 5 × 10^6^ mL^−1^ cell achieved. Similarly, the hydrogel mixture with 5% GelMA and 5 × 10^6^ mL^−1^ PS fluorescent particles was also made.

### Characterization of Cells (Particles) Patterns in Cured Hydrogel

The samples were viewed under a fluorescence microscope (BZ‐X800, Keyence, Japan). Hoechst 33342 (Solarbio) was used to label the cells for 20 mins in the 37 °C water bath. For live/dead viability assay, the cells encapsulated in the hydrogel were dyed in solution which consists of 2 mL PBS solution, 4 µL 2 mm EthD‐1 solution, and 1 µL 4 mm calcein AM solution.

### Cell Toxicity, and Viability Tests

The cell viability was tested using a non‐destructive bioluminescent cytotoxicity assay of Toxilight bioassay (LT17‐217, Lonza, Rockland, USA), which measured the release of AK from damaged cells. First, the samples of cells encapsulated in hydrogel fibers were cultivated in a petri dish with DMEM high sugar complete medium (SNM‐002E, SUNNELL) in a 5% CO_2_ incubator at 37 °C for 6, 24, 48, 72 h, respectively. Then, 3 mL supernatant sampling was collected from the petri dish to the centrifuge tube for cell viability tests. After that, all experiments were performed according to the manufacturer's protocols using the same volume of different samples.

## Conflict of Interest

The authors declare no conflict of interest.

## Supporting information

Supporting Information

## Data Availability

The data that support the findings of this study are available from the corresponding author upon reasonable request.
