# Supplementary material for: Acoustic Cell Patterning for Structured Cell‐Laden Hydrogel Fibers/Tubules
Source: Adv Sci (Weinh). 2024 Feb 2;11(14):2308396. doi: 10.1002/advs.202308396 (PMC11005686; doi:10.1002/advs.202308396)
Supplement: Supplementary file 1 — Supporting Information [file ADVS-11-2308396-s001.pdf]

ADVANCED SCIENCE
Open Access

Supporting Information

for *Adv. Sci.*, DOI 10.1002/advs.202308396

Acoustic Cell Patterning for Structured Cell-Laden Hydrogel Fibers/Tubules

Qiu Yin, Yucheng Luo, Xianglin Yu, Keke Chen, Wanlu Li, Hu Huang, Lin Zhang, Yinning Zhou, Benpeng Zhu, Zhichao Ma and Wenming Zhang**

Supplementary Materials for

Acoustic cell patterning for structured cell-laden hydrogel fibers/tubules

Qiu Yin[1, 2], Yucheng Luo[2], Xianglin Yu[3], Keke Chen[2], Wanlu Li[4], Hu Huang[5], Lin Zhang[6], Yinning Zhou[7], Benpeng Zhu[8], Zhichao Ma[2], Wenming Zhang[1,3]**

1. State Key Laboratory of Mechanical System and Vibration, Shanghai Jiao Tong University, Shanghai 200240, China

2. Institute of Medical Robotics, School of Biomedical Engineering, Shanghai Jiao Tong University, No.800 Dongchuan Road, Shanghai 200240, China

3. SJTU Paris Elite Institute of Technology, Shanghai Jiao Tong University, Shanghai 200240, China

4. School of Biomedical Engineering and Med-X Research Institute and Shanghai Jiao Tong University Shanghai 20030, P. R. China

5. Key Laboratory of CNC Equipment Reliability, Ministry of Education, School of Mechanical and Aerospace Engineering, Jilin University, Changchun, Jilin 130022, China

6. School of Mechatronic Engineering, Changchun University of Technology, Changchun 130012, China

7. Joint Key Laboratory of the Ministry of Education, Institute of Applied Physics and Materials Engineering, University of Macau, Avenida da Universidade, Taipa, Macau, 999078, China

8. School of Integrated Circuit, Wuhan National Laboratory for Optoelectronics, Huazhong University of Science and Technology, Wuhan, China

**Correspondence: wenmingz@sjtu.edu.cn (W.M. Zhang); zhichaoma@sjtu.edu.cn （Z.C. Ma）*

This PDF file includes:

Fig. S1 to S2

Tables S1

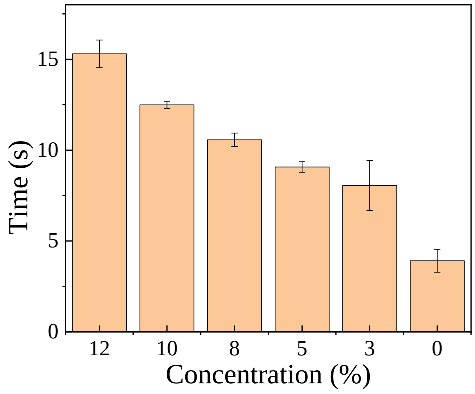

Fig.S1 The correspondences between cell arrangement time and different GelMA concentrations.

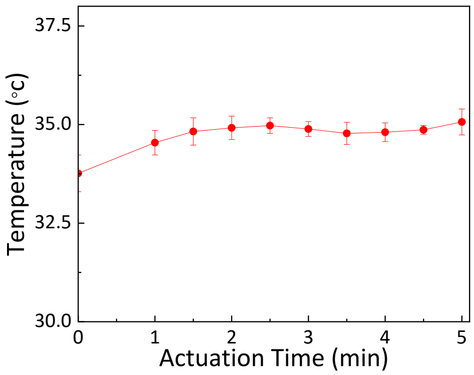

Fig.S2 The temperature variation with working time of PZT transducer

Table S1. Comparison of different biofabrication techniques for cell arrangement

Cell arrangement method	Principle	Resolution	Dimension	Speed	Dynamic tunability	Ref.
Hydrogel mold patterning	Using hydrogel macrofilaments as the structure mold	Moderate (300-500 um)	3D	Low (line-by-line dispensing)	High	[1, 2]
Topographical patterning	Cells are seeded on the substrates with different topographical structures prepared using micro fabrication technology	High (nano-to micro-scale)	2D	Low (non-continuous production)	Low	[3, 4]
Microfluidic spinning with pattern structure	Introducing a sheath fluid with a high-speed flow rate in a microfluidic platform with grooves, in which the alginate solution could be both flattened and polymerized	High (less than 100 um)	3D	High (continuous production of fibers)	Low	[5]
Surface chemical treating	Introducing patterned biological molecules in the substrate by microfabrication to induced the cell arrangement	High (less than 100 um)	2D	Low (non-continuous production)	Low	[6-8]
Electric activating	Cells are arranged by the dielectrophoresis (DEP) trap	High (less than 100 um)	2D	Low (non-continuous productio	Low	[9, 10]

				n)		
Magnetic activating	Magnetically labeled cells align via designed magnetic field	Low	3D	Low (non-continuous production)	Low	[11, 12]
Acoustic activating (Present work)	Cells are arranged by the designed acoustic pressure field	Moderate (less than 500 um)	3D	High (continuous production of fibers/tubules)	High	

References:

[1] C. Norotte, F.S. Marga, L.E. Niklason, G. Forgacs, Scaffold-free vascular tissue engineering using bioprinting, Biomaterials 30(30) (2009) 5910-5917.

[2] A. Skardal, J. Zhang, G.D. Prestwich, Bioprinting vessel-like constructs using hyaluronan hydrogels crosslinked with tetrahedral polyethylene glycol tetracrylates, Biomaterials 31(24) (2010) 6173-6181.

[3] J. Yang, F.R.A.J. Rose, N. Gadegaard, M.R. Alexander, A High-Throughput Assay of Cell-Surface Interactions using Topographical and Chemical Gradients, Adv. Mater. 21(3) (2009) 300-304.

[4] M.T. Lam, S. Sim, X. Zhu, S. Takayama, The effect of continuous wavy micropatterns on silicone substrates on the alignment of skeletal muscle myoblasts and myotubes, Biomaterials 27(24) (2006) 4340-4347.

[5] E. Kang, Y.Y. Choi, S.-K. Chae, J.-H. Moon, J.-Y. Chang, S.-H. Lee, Microfluidic Spinning of Flat Alginate Fibers with Grooves for Cell-Aligning Scaffolds, Adv. Mater. 24(31) (2012) 4271-4277.

[6] C.-C. Lin, C.C. Co, C.-C. Ho, Micropatterning proteins and cells on polylactic acid and poly(lactide-co-glycolide), Biomaterials 26(17) (2005) 3655-3662.

[7] C. Williams, A.W. Xie, M. Yamato, T. Okano, J.Y. Wong, Stacking of aligned cell sheets for layer-by-layer control of complex tissue structure, Biomaterials 32(24) (2011) 5625-5632.

[8] D. Martinez, C. Py, M. Denhoff, R. Monette, T. Comas, A. Krantis, G. Mealing, Polymer peel-off mask for high-resolution surface derivatization, neuron placement and guidance, Biotechnology and Bioengineering 110(8) (2013) 2236-2241.

[9] C.-T. Ho, R.-Z. Lin, R.-J. Chen, C.-K. Chin, S.-E. Gong, H.-Y. Chang, H.-L. Peng, L. Hsu, T.-R. Yew, S.-F. Chang, C.-H. Liu, Liver-cell patterning Lab Chip: mimicking the morphology of liver lobule tissue, Lab Chip 13(18) (2013).

[10] J. Ramon-Azcon, S. Ahadian, M. Estili, X. Liang, S. Ostrovidov, H. Kaji, H. Shiku, M. Ramalingam, K. Nakajima, Y. Sakka, A. Khademhosseini, T. Matsue, Dielectrophoretically aligned

carbon nanotubes to control electrical and mechanical properties of hydrogels to fabricate contractile muscle myofibers, Adv Mater 25(29) (2013) 4028-34.
[11] S. Richard, A.K.A. Silva, G. Mary, H. Ragot, J.E. Perez, C. Ménager, F. Gazeau, I. Boucenna, O. Agbulut, C. Wilhelm, 3D Magnetic Alignment of Cardiac Cells in Hydrogels, ACS Applied Bio Materials 3(10) (2020) 6802-6810.
[12] N. Demri, S. Dumas, M.L. Nguyen, G. Gropplero, A. Abou-Hassan, S. Descroix, C. Wilhelm, Remote Magnetic Microengineering and Alignment of Spheroids into 3D Cellular Fibers, Advanced Functional Materials 32(50) (2022).
